# Family planning for urban slums in low- and middle-income countries: a scoping review of interventions/service delivery models and their impact

**DOI:** 10.1186/s12939-021-01518-y

**Published:** 2021-08-19

**Authors:** John Kuumuori Ganle, Leonard Baatiema, Paul Ayamah, Charlotte Abra Esime Ofori, Edward Kwabena Ameyaw, Abdul-Aziz Seidu, Augustine Ankomah

**Affiliations:** 1 Department of Population, Family and Reproductive Health, School of Public Health, University of Ghana, P. O. Box LG 13 Legon, Accra, Ghana; 2 Department of Health Policy, Planning and Management, School of Public Health, University of Ghana, Accra, Ghana; 3 AIDS Commission, Accra, Ghana; 4 Regional Institute for Population Studies, University of Ghana, Legon, Accra, Ghana; 5 School of Public Health, Faculty of Health, University of Technology Sydney, NSW Sydney, Australia; 6 Department of Population and Health, University of Cape Coast, Cape Coast, Ghana; 7 College of Public Health, Medical and Veterinary Sciences, James Cook University, Townsville, Queensland Australia; 8 Population Council, Accra, Ghana

**Keywords:** Family planning, Contraception, Reproductive health, Service delivery, Urban slums, Scoping review

## Abstract

**Background:**

Although evidence suggest that many slum dwellers in low- and middle-income countries have the most difficulty accessing family planning (FP) services, there are limited workable interventions/models for reaching slum communities with FP services. This review aimed to identify existing interventions and service delivery models for providing FP services in slums, and as well examine potential impact of such interventions and service delivery models in low- and middle-income settings.

**Methods:**

We searched and retrieved relevant published studies on the topic from 2000 to 2020 from e-journals, health sources and six electronic databases (MEDLINE, Global Health, EMBASE, CINAHL, PsycINFO and Web of Science). Grey and relevant unpublished literature (e.g., technical reports) were also included. For inclusion, studies should have been published in a low- and middle-income country between 2000 and 2020. All study designs were included. Review articles, protocols or opinion pieces were excluded. Search results were screened for eligible articles and reports using a pre-defined criterion. Descriptive statistics and narrative syntheses were produced to summarize and report findings.

**Results:**

The search of the e-journals, health sources and six electronic databases including grey literature and other unpublished materials produced 1,260 results. Following screening for title relevance, abstract and full text, nine eligible studies/reports remained. Six different types of FP service delivery models were identified: voucher schemes; married adolescent girls’ club interventions; Willows home-based counselling and referral programme; static clinic and satellite clinics; franchised family planning clinics; and urban reproductive health initiatives. The urban reproductive health initiatives were the most dominant FP service delivery model targeting urban slums. As regards the impact of the service delivery models identified, the review showed that the identified interventions led to improved targeting of poor urban populations, improved efficiency in delivery of family planning service, high uptake or utilization of services, and improved quality of family planning services.

**Conclusions:**

This review provides important insights into existing family planning service delivery models and their potential impact in improving access to FP services in poor urban slums. Further studies exploring the quality of care and associated sexual and reproductive health outcomes as a result of the uptake of these service delivery models are essential. Given that the studies were reported from only 9 countries, further studies are needed to advance knowledge on this topic in other low-middle income countries where slum populations continue to rise.

**Supplementary Information:**

The online version contains supplementary material available at 10.1186/s12939-021-01518-y.

## Introduction

Globally, people living in informal settlements (slums) in urban areas will reach 2 billion by 2030 [1]. The UN defines slums as areas with high population density, which lack one or more of the following conditions: access to improved water, access to improved sanitation, sufficient living area, housing durability, and security of tenure [2]. It is estimated that, in 2015, the proportion of urban populations living in slums in different regions of the world were as follows: Northern Africa (11.9 %); Sub-Saharan Africa (55.9 %); Latin America and the Caribbean (21.1 %); Eastern Asia (26.2 %); Southern Asia (31.3 %) South-Eastern Asia (28.4 %); Western Asia (24.9 %); and Oceania. (24.1 %) [2].

In Ghana, an estimated 37.9 % (5.4 million) of urban dwellers live in slums [3]. Within current literature, there is consensus that many slum neighbourhoods in Africa are precarious spaces of human habitation [4–10]. For instance, slums are often characterized by poor infrastructure, poverty, and violence in addition to frequently not being recognized by public authorities as integral parts of cities [7, 11–13]. This disadvantage transcends several spheres to include poor access to family planning (FP) and contraceptive services [6, 14, 15]. In broad terms, family planning is the voluntary planning and action taken by individuals to prevent, delay or achieve a pregnancy [16]. The ultimate aim of family planning is to help people decide freely and responsibly on the number of children they want to have and when to have them [16]. Thus, family planning enables couples and individuals to delay pregnancy, space births, limit family size, prevent unintended pregnancies and sexually transmitted infections (STIs) including HIV and AIDS, as well as helps couples and individuals who want to have children achieve their desires [16]. In this study, family planning services considered included family planning information provision, family planning counselling, contraceptive services, post-abortion family planning services, and STI screening, prevention and treatment services.

Current scholarly and programmatic literature on reproductive health suggest that many slum dwellers in low-income settings have the most difficulty accessing sexual and reproductive healthcare [6, 7, 11, 13]. In addition, slum environments are often characterized by high levels of substance use, early sex, transactional sex and age asymmetry of sexual partners as well as high rates of sexual and gender-based violence [6, 13]. These conditions potentially expose women and adolescent girls in slums to high risks of unplanned pregnancies [13].

A number of factors have been identified to contribute to poor access to family planning services in slums. First, many slum residents are often ‘time poor’, and this reduces their ability to access services [13]. Poor slum residents may have to travel outside their neighbourhoods for high quality and free or low-cost family planning services because government services in the vicinity of informal settlements are often in poorer state and lack key supplies than clinics in other parts of cities [13]. Where slum dwellers are served at all, they are often served by private informal providers [13]. Besides, in some countries private providers in urban slums may also be poorly regulated and may not be well integrated into the public health sector [11, 13]. Private services in urban slums may also be of poor quality and lacking basic facilities and supplies [11]. Private providers may even charge higher user fees for contraceptives. Also, misinformation and rumours can undermine use of contraceptives even in areas with high unmet need for FP [11]. In addition, the localized networks of slum dwellers may further reduce their awareness and knowledge about contraceptive methods and services. The combined effect of this multiple deprivation is high unmet need for FP services [10, 11].

Over the years, the precarious conditions in slums have attracted a number of research and policy analyses. Slum populations have also attracted specially designed interventions to improve access to, and uptake of, quality and affordable FP services. However, to the best of our knowledge, no review has been conducted to pull together the disparate literature in order to better understand what FP service delivery models/interventions currently exist and their impact. This knowledge gap could potentially hinder efforts to improve not just reproductive health outcomes in slums but also overall sustainable urban development as envisaged under sustainable development goal (SDG) 11. As previous researchers have noted, poor access to FP services could negatively affect overall urban development and governance via high fertility, rapid population growth and urbanisation, increased demand for urban services, and poor health outcomes [13]. The aims of this scoping review were (1) to identify existing interventions and service delivery models for providing FP services to slum communities; and (2) to assess their actual and potential impact.

## Methods

This scoping review was conducted according to Arksey and O’Malley’s framework on evidence synthesis [17]. The framework proposed five key steps in iteratively generating and synthesizing evidence: (1) identification of key research questions(s); (2) identification of relevant eligible studies; (3) selection of eligible studies; (4) data charting and; (5) collating, summarizing and reporting of results. To ensure rigour and transparency in reporting, this review was conducted according to the Preferred Reporting Items for Systematic reviews and Meta-Analyses extension for Scoping Reviews (PRISMA-ScR).

### Review questions

The scoping review was conducted to answer the following questions: what FP interventions and/ or service delivery models currently exist in urban slums in low- and middle-income settings; and what has been their potential/actual impact in terms of improving access to family planning services? To effectively answer these questions, a scoping review was favoured over a systematic review because the strict inclusion and reporting criteria of a systematic review would make it impossible to incorporate otherwise useful materials such as unpublished reports on potential impact of FP interventions. In other words, the methodological approach to a scoping review is relatively flexible and allows for the inclusion of diverse study designs and grey literature.

### Identifying eligible studies

A search strategy was developed to facilitate the search and selection of relevant studies to address the two research questions on existing interventions and service delivery models and their impact. The search sources comprised E-journals, health sources and six electronic databases including MEDLINE, Global Health, EMBASE, CINAHL, PsycINFO and Web of Science. Grey and unpublished literature were also searched for relevant studies.

The literature search process was in three phases. The first comprised a preliminary search of selected databases to analyze relevant text words on the subject matter reported in potentially eligible studies. The second search involved electronic search of each of the selected databases as outlined above using a pre-tested search strategy. The search strategy was modified according to each database using the same key search terms and medical subject headings (MeSH) terms. The third round of search was through manual hand-searching of the reference lists of selected articles. Supplementary file 1 outlines the MEDLINE database search strategy, which was modified and used for each database where appropriate.

For inclusion, studies should have been published in a low- and middle-income country between 2000 and 2020, a time frame to ensure that the eligible studies on existing interventions are current and relevant to address the family planning needs of populations in urban slums. Only articles published in the English language were considered. All study designs were included. For inclusion, studies were original research articles with focus on FP interventions or programmes in urban slums in low- and middle-income countries. Where relevant, the review also considered reports, policy documents, and working papers. Review articles, protocols or opinion pieces were excluded though reference may be made to some of them in the main work. The review also included grey literature relevant to the review topic.

### Study selection

**Fig. 1:**
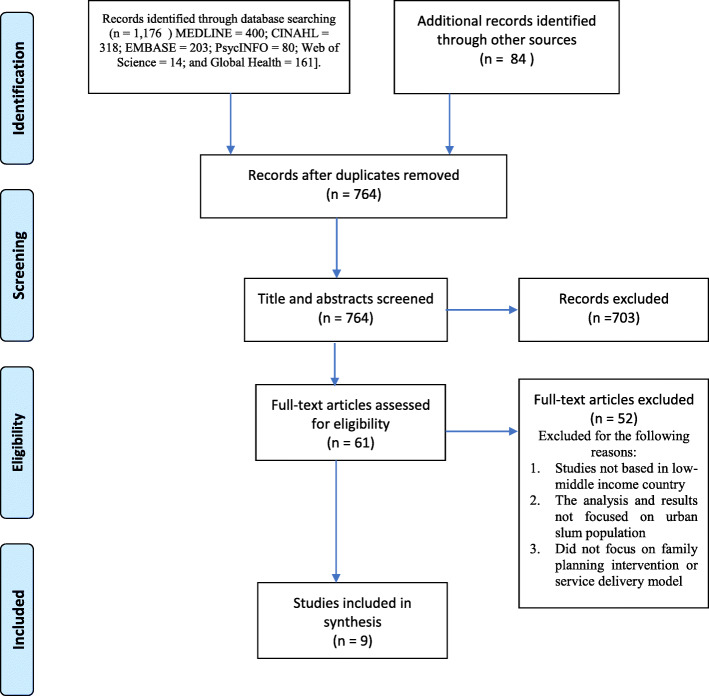
Evidence Flow Chart

All databases were searched individually, and studies imported into EndNote. Selection of potentially eligible studies proceeded as follows. First, duplicates of studies from all databases search results were removed. Second, titles of articles were assessed by two reviewers (JGK & LB) for relevance using the study inclusion and exclusion criteria. The third round involved review and screening of abstracts of retained studies after title relevance screening. In the fourth stage, full text of articles was reviewed for relevance and where eligibility criteria were met, articles were selected for final analysis. Six of the authors (LB, JGK, PA, CAEO, EAN and AA), of this review paper independently assessed the full text of these articles to evaluate their potential eligibility. Articles from reference lists which met the inclusion criteria were also screened and eligible studies included in the final analysis. A search decision flowchart detailing the search process at every stage including duplicates removal, exclusions at title assessment, abstract and full-text screening and inclusions after reference screening is shown in Fig. 1.

### Extracting and charting the results

A standardized, pre-tested data extraction form was developed and reviewed by the team to ensure that all relevant information in relation to answering the review questions were captured. In brief, the data extraction form captured data on the authors, year of publication, country of study, study designs, aims/objectives of the study, study population, sampling and sample size, methods, type of intervention, duration of intervention and key study findings. Key study outcomes or findings related to any direct effects of the implemented family planning intervention or service delivery model were reported.

### Collating, summarizing and reporting of results

Following extraction of the data into an evidence table, we summarized the main studies and displayed in a chart to show study locations, FP interventions and barriers to accessing FP services in urban slums. In reporting the study findings, we adopted the narrative synthesis approach, a widely used approach to reporting scoping review findings [18]. This approach is particularly suitable for reporting findings from eligible studies with different study designs [19].

## Results

### Overview of studies

The search generated a total of 1,176 peer reviewed articles [MEDLINE = 400; CINAHL = 318; EMBASE = 203; PsycINFO = 80; Web of Science = 14; and Global Health = 161]. Search from other sources including grey literature and published reports resulted in 84 studies. The total number of articles or reports was thus 1,260. A total of 496 duplicates were removed and the rest (764) remained for title relevance and abstract screening. Following screening of title and abstract for study relevance, a further 703 were removed and 61 papers were considered for full-text screening. Following full-text screening, only 9 articles and grey reports were considered eligible for the review.

### Characteristics of eligible studies

The included studies were conducted in 9 different low-income countries and low-and middle-income countries, namely Bangladesh, Ghana, Nigeria, Uganda, Kenya, Senegal, India, Pakistan, and Nicaragua. The studies were published between 2005 and 2020. The types of FP services reported in the study comprised condoms; injectable contraceptives; IUDs; tubectomy; vasectomy; pill; sterilization; emergency contraception; oral contraceptives; and implants.

The study designs employed in the eligible studies were mixed. For example, most were quasi/experimental in design [20–23]; one was a retrospective study [24], a cross-sectional study [25], a technical report [26] and the rest were evaluation/baseline studies [27, 28]. A summary of the study characteristics is reported in Table 1.

**Table 1:** Characteristics of eligible studies on family planning interventions in urban slums

#	Country	Authors, publication year	Study Aim	Study design	Family planning services	FP service delivery model & intervention	Population, sample, age	Data collection methods
1	Bangladesh	Huda et al. 2019 [21]	To assess the effectiveness of a married adolescent girls club in reducing the unmet need for family planning (FP)	Quasi-experimental study	Condom, injection, IUD, tubectomy, vasectomy, implant and pill	Married Adolescent Girls’ (MAG) club intervention	Married adolescent girls Club Sample = 1601 Age = 14–19	quantitative survey
2	Pakistan	Hennink and Clements, 2005 [20]	To determine the impact of new family planning clinics on knowledge, contraceptive use, and unmet need for family planning among married women in poor urban areas	Quasi-experimental study	Pill, condoms, injectables, the IUD, female sterilization procedures), pregnancy testing, termination of pregnancy, and advice about sexual health.	Franchised Family Planning Clinics	Age: <20–40+	Interviews; descriptive statistics,
3	Nicaragua	Meuwissen et al. 2006 [25]	to identify the nature of existing unmet needs for SRH care through voucher redemption	Cross-sectional study	Oral, injectables, IUD and condoms	Voucher scheme	Sample: 3301 Age: 11–20	Structured questionnaire, descriptive statistics; multivariable analysis
4	India	Achyut et al., 2016 [27]	To evaluate the impact of the Urban Health Initiative	Evaluation study (longitudinal sample of women and health facilities with baseline (2010) and endline (2014) data)	Sterilization (female or male) IUD Oral contraceptive pill Condom Other modern method	Urban reproductive health initiative:	women of reproductive age (15–49) women and the sub-sample of poor women	interviewer administered facility audit, provider interviews and exit interviews
5	Senegal	Benson et al. 2018 [28]	To examine the impact of the Initiative’s demand- and supply-side activities on modern contraceptive use.	Evaluation Baseline (2011) and endline (2015) longitudinal data	Sterilization (female or male) IUD Oral contraceptive pill Condom Other modern method	Urban reproductive health initiative:	women of reproductive age (15–49) women and the sub-sample of poor women	interviewer administered facility audit, provider interviews and exit interviews
6	Kenya & Uganda	Arur et al., 2009 [26]	To improve use, responsiveness, and quality of FP and Safe Motherhood services and give clients a choice of providers.	Technical report (review from published and unpublished secondary literature, primary data collection, and interviews)	Implants, female sterilization, intrauterine contraceptive devices (IUDs)	Voucher scheme	Poor women in urban centres	secondary literature, primary data collection, and extensive interviews
7	Nigeria	Krenn et al. 2014 [22]	To determine the contribution of mobile services to total family planning services	Quasi-experimental study	N/A	Nigerian Urban Reproductive Health Initiative	Men and women of reproductive age	Interviews, secondary data analysis, descriptive statistics
8	Ghana	Henry et al., 2020 [24]	To generate estimates of the effect of the Willows home-based counselling model as implemented in Kumasi, Ghana from 2013 to 2016 in order to guide future programming for community-based family planning behaviour-change interventions in urban Ghana and similar West African settings.	Retrospective, cross-sectional design	Male or female sterilization, intrauterine device, implants, injectables, oral contraceptive pills, male or female condoms, lactational amenorrhoea method and emergency contraception.	The Willows reproductive health programme	women who were between the ages of 16 and 44 in 2013 1836 women in each of the intervention and comparison areas, which we rounded up to a sample of 2000 women in each site.	Retrospectively assessed changes in women’s contraceptive use Household survey
9	Bangladesh	Uddin et al. 2012 [23]	To assess the effectiveness of two models to provide primary healthcare (PHC) services to street-dwellers.	Experimental pre-post design	Condom, pill, and injection	Static clinic and satellite clinics	800 (400 females and 400 males) street-dwellers, ever-married females and males aged 15 years and above, living within the two-kilometre radius of the study locations; and were sleeping in the area for at least one week before data-collection.	Mixed method approach, a combination of both quantitative and qualitative techniques, was used for data-collection. The community survey and qualitative components (in-depth interviews with study subjects and healthcare providers)

### Family planning interventions and service delivery models

**Fig. 2:**
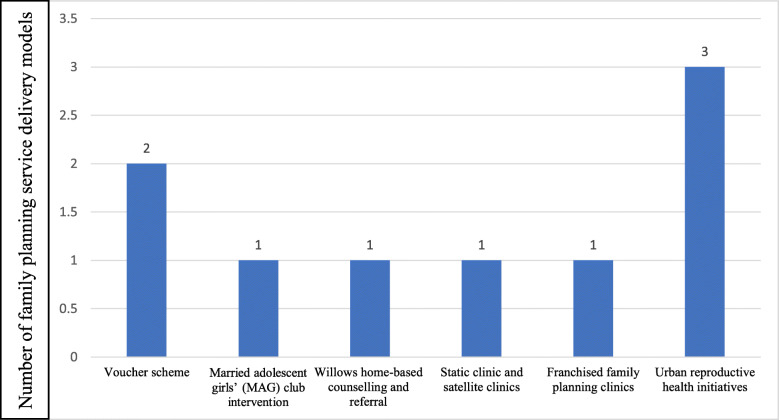
Distribution of FP service delivery models for urban slums in low/middle-income countries

The studies reported six different types of service delivery models or interventions to improve access to FP services in urban slums (see Fig. 2). These included (1) voucher schemes [25, 26, 29]; (2) married adolescent girls’ club intervention [21]; (3) franchised FP clinics [20]; (4) urban reproductive health demand-creation and supply programmes [22, 27, 28]; (5) Willows home-based counselling and referral programme [24]; and (6) Static clinic and satellite clinics [23]. Details of the study characteristics are summarised in Table 1. These interventions and/ or service delivery models are described in detail below.

#### Voucher schemes and access to FP services

Distribution of vouchers for accessing free sexual and reproductive health services was identified as a service delivery model to improve access to FP services in poor urban slums. Two separate studies reported the use of this service delivery model [25, 26].

The first was a cross-sectional study conducted in Nicaragua to identify the nature of existing unmet needs for SRH care through voucher redemption between September 2000 and July 2001 [25]. This scheme provided, at no cost, uninhibited access to sexual and reproductive healthcare in 19 primary healthcare facilities in Managua. The vouchers were distributed through the Central American Health Institute (ICAS), NGOs, project clinics, four markets, 19 public schools and 22 poor neighbourhoods. Female adolescents were mainly the distributors of the vouchers and vouchers were valid for three months. The voucher package for beneficiaries comprised free-of-charge consultation and a follow-up visit for advice/counselling, contraception, treatment of STIs or reproductive tract infections, pregnancy testing and/ or antenatal care in any of the four public, five private and 10 NGO clinics. The clinics received reimbursement for each adolescent consultation. During each consultation, doctors completed standardized clinical forms. Voucher redeemers received a booklet on adolescent health, two condoms with supportive information, and if required, contraceptive methods, laboratory tests and syndromic treatment for STIs.

The second voucher scheme, which comprised the Reproductive Health Voucher Project (RHVP) and the Reproductive-Health Output-Based Approach (RH-OBA) voucher pilot programme, were implemented in Uganda and Kenya respectively [26]. The RH-OBA project was implemented in three rural districts and two slums in Nairobi for eligible beneficiaries to purchase FP vouchers for both long and permanent methods at $1.25 and safe motherhood voucher for antenatal, institutional delivery and postnatal care services at $2.50. The RH-OBA provides safe motherhood services at $1.50. Marie Stopes International Uganda was appointed as the Voucher Management Agency. A key feature of the RHVP scheme was that FP services vouchers could only be redeemable at private for-profit and non-profit providers.

#### Married adolescent girls’ (MAG) club intervention

One study was identified, which reported use of a club-based service delivery model in Bangladesh [21]. This was a quasi-experimental study conducted in four urban slums in 2016 to examine the effectiveness of MAG in reducing unmet needs for FP among married girls between the ages of 14 and 19. The MAG intervention was developed and implemented to tackle the unmet FP needs of adolescents in slums in Bangladesh. This MAG was rolled out in response to early marriage and childbearing often resulting in pregnancy complications, spousal violence and low-birth weight of babies in urban slums in Bangladesh. The MAG targets married adolescent girls between 14 and 19 years’ old, both pregnant and non-pregnant and living with their husbands and/or families in four selected urban slums in Bangladesh. The MAG held monthly sessions covering 40 sessions under the guidance of facilitators recruited on the project. Some of the activities undertaken as part of the MAG to its members comprised dance, music and drama sessions. Services provided under the MAG model comprised comprehensive information and services on FP. To assess the effectiveness of the club-based model, an intervention and control groups were set up comprising 799 participants for the intervention group (areas) and 802 for the control groups (areas). These participants were drawn from 10 clusters each from the intervention and control groups.

#### Franchised family planning clinics

The use of socially franchised clinics for FP was identified as one of the service delivery models for urban poor communities. The study was commissioned against the background of high fertility rates and unmet needs for FP services in Pakistan. Using a quasi-experimental design, the study aimed to examine the impact of new family planning clinics on knowledge, contraceptive use, and unmet need for family planning among married women aged 15–45 years in poor urban areas of six secondary cities of Pakistan [24]. The services provided as part of this project comprised contraceptives (pills, condoms, injectables, IUD, female sterilization), pregnancy testing, pregnancy termination and advice about sexual health. Both outreach and clinic-based services were utilized, and the latter was facilitated by teams of community-based distributors. Fees charged for the services provided at the clinics were relatively lower than the cost at private health facilities due to the subsidized treatment funds available to the urban poor community members as part of the franchising FP clinic intervention. In the study, baseline and end line surveys were conducted to collect data from a population of 5,338 for the baseline and 5,502 for the endline in four intervention sites and two control sites. The study collected data on knowledge of modern contraception, contraceptive prevalence, and unmet need for FP.

#### Urban reproductive health demand-creation and supply programmes

Three studies were identified which reported on interventions seeking to improve uptake of FP services in poor urban communities [22, 27, 28]. The first study was conducted in a Senegalese context where uptake and use of FP services was low, and the prevalence of modern contraceptive use was only about 20 % [28]. The urban reproductive health initiative is an integrated, multi-dimensional project which seeks to address this problem among women. The project had both demand-side and supply-side components. The former recruited, trained and used Muslim religious leaders to become FP champions, to promote FP messages in religious settings, and debate and campaign for access to FP services in the media (radio, television and print media). As part of the intervention, community-health volunteers were recruited and trained to share information about FP through individualized discussions with women and other members of the household. The supply-side component sought to ensure sufficient and regular supply of FP services in the project sites, improve access to quality FP services, and strengthen the referral system. A cross-sectional study was subsequently conducted in all the six cities in Senegal to evaluate the impact of this initiative using a 2011 baseline and 2015 endline longitudinal data. There was an increase in contraception use between baseline and endline. First, and for all women, modern and traditional contraception use rose from 16.9 to 2.0 % at baseline to 22.1 and 2.3 % at endline respectively. Second, results for poor women and less poor women were similar. Third, the proportions of non-users saw a decreased from 81.1 to 75.5 % for all women and 81.4–73.4 % for poor women.

The second study was an Urban Health Initiative, which aimed to increase FP use among poor women in urban areas of Uttar Pradesh, India [27]. Against the context of low uptake of FP services among the poor, access to low quality FP services and high maternal and child mortalities, the project was designed to increase the use of FP services by the urban poor, improve quality of FP services, reduce maternal and infant mortality. Key components of the project comprised provision of postpartum and post abortion FP, delivery of training to FP providers to improve their technical competence and client-provider interactions, expansion of the role of the private sector in FP service provision, use of community health workers for outreach activities and use of mass-media to promote demand for FP services. Community health workers were tasked to visit homes within slums to offer education on FP methods and offer counselling on postpartum FP; accompany women to a health facility; refer women to a health facility on fixed service days; and where necessary, provide short-term methods (pills and condoms) to interested parties.

The third study was the Nigerian Urban Reproductive Health Initiative (NURHI) – a 6-year comprehensive family planning programme (2009–2015) in 4 cities (Abuja, Ibadan Ilorin and Kaduna) [22]. The project adopted multiple communication pathways to facilitate dialogue on family planning, increase social approval for it, and improve accurate knowledge about contraceptives. As part of the project, mobile service delivery was later introduced to improve access to clinical methods in slums. This study analyzed data from representative baseline (2010–11) and midterm (2012) surveys of women of reproductive age in the project cities. The findings showed that, between baseline and midterm, the percentage of women who believed in myths or had misconceptions about contraception declined by between 9 and 17 % points on outcomes measured. Intention to use contraception in the next 12 months also recorded an increase of between 7.5 and 10.2 % points in all four cities. The prevalence of contraception use increased by between 2.3 % points (in Abuja) and 15.5 % point (in Kaduna) from baseline to midterm. The study noted that several of the NURHI communication interventions were significantly associated with higher levels of contraceptive use, and propensity score matching found a 9.9 % point increase in contraceptive use in the 4 cities attributable to project exposure.

#### Willows home-based counselling and referral programme

The Willows reproductive health intervention was a 3-year home-based counselling and referral programme for women in low-income urban settlements in the Ashanti region of Ghana [24]. The intervention involves information sharing and education, including counselling, and referral on FP. As part of the programme, visits are undertaken, counselling and referral targeting 20,000–50,000 women of reproductive age are done. As part of the intervention, field educators are recruited, trained and deployed to conduct home visits, create awareness about FP services, and refer women to locally based health services. A retrospective analysis of changes in women’s contraceptive use over the 5 years prior to the survey, including before the Willows programme launch, at the end of the programme, and 18 months after the close of the programme was subsequently conducted. Overall, the intervention resulted in a 10.5 %-point increase in use of modern contraceptives from baseline to endline (95 %CI: 6.2, 14.8; *P* < 0.001) and a 7.6 % point increase from baseline to follow-up (95 %CI: 3.3, 11.9; *P* < 0.001). The programme had a significant impact on modern contraceptive use at end line among women who received some information or counselling visit. However, only 20.2 % of women in the Willows intervention area reported a visit, hence the intervention did not achieve its aim to reach all reproductive-aged women in the community.

#### Static clinic and satellite clinics

The static clinic and satellite clinic intervention was evaluated through an experimental pre-post design study that tested two models: static clinic and satellite clinics, for providing primary healthcare services to street-dwellers through paramedics in Dhaka city from May 2009 to April 2010 using both quantitative and qualitative techniques [23]. An analysis of the longitudinal data shows that the use of healthcare services by female street-dwellers increased by 56 and 31 % between baseline and end line in both the model clinic areas, and the difference was highly significant (*p* < 0.001). The study showed a significant increase in the use of family planning methods among females at end line compared to baseline in both the static and satellite clinics. Use of family planning methods recorded a significant increase among males also at endline compared to baseline in the static clinic. The use of semi-permanent and permanent methods among males remained almost same at endline in model clinic 1 areas while it increased in model clinic 2 areas (Table 2).

**Table 2:** Summary of evidence on impact of FP interventions/ service delivery models for urban slums

Country	Author	FP intervention	Outcomes measured	Impact of family planning service delivery model
Bangladesh	Huda et al. 2019 [21]	Married Adolescent Girls Club	Effectiveness of a married adolescent girls club in reducing unmet need for family planning	• The percentages of the targeted population using any modern method of contraception were significantly higher among respondents in the intervention areas than those in the control areas (72.6 % versus 63.5 %). • The unmet need for FP was significantly lower among respondents in the intervention areas than that of the control areas (16.2 % versus 20.7 %). • The MAG club was a well-received strategy to provide comprehensive information on FP, which in turn helped improve contraceptive method practices and reduced the unmet need for FP among married adolescent girls in urban slums in Bangladesh
Pakistan	Hennink and Clements, 2005 [20]	Franchised family planning clinics	Knowledge, contraceptive use and unmet needs for family planning services	• The clinics contributed to a 5 % increase in overall knowledge of family planning methods and an increase in knowledge of female sterilization and IUD of 15 and 7 % respectively. • Distinct effects were found on contraceptive uptake, including an 8 % increase in female sterilization and a 7 % decline in condom use. • Unmet need for family planning declined in two sites, whereas impacts on the other sites were variable. • Although the new clinics are located within poor urban communities, users of the services were not the urban poor, but rather were select sub-groups of the local population.
Senegal	Benson et al. 2018 [28]	Urban Health Initiative	Impact of demand and supply-side activities on modern contraceptive use	• By endline there was increased exposure to radio and television programming, religious leaders speaking favourably about contraception, and community-based initiatives. • In the same period, modern contraceptive use increased from 16.9–22.1 % with a slightly larger increase among the poor (16.6–24.1 %) • Multivariate analysis demonstrate that women exposed to community-based activities were more likely to use modern contraception by end line (marginal effect (ME): 5.12; 95 % confidence interval (CI): 2.50–7.74) than those not exposed. • Further, women living within 1 km of a facility with family planning guidelines were more likely to use (ME: 3.54; 95 % CI: 1.88–5.20) than women without a nearby facility with guidelines. • Among poor women, community-based activities, radio exposure (ME:4.21; 95 % CI: 0.49–7.93) and living close to program facilities (ME: 4.32; 95 % ci: 0.04–8.59) impacted use.
India	Achyut et al., 2016 [27]	Urban Health Initiative	Impact of demand and supply side factors influencing access to and provision of FP	• Impact evaluation results show significant effects of exposure to both demand and supply side program activities. • In particular, women exposed to brochures (marginal effect: 6.96, pb.001), billboards/posters/wall hangings (marginal effect: 2.09, pb.05), and FP on the television (marginal effect: 2.46, pb.001) were significantly more likely to be using a modern method at end line. • In addition, borderline significance for being exposed to a community health worker (marginal effect: 1.66, pb.10) and living close to an improved public and private supply environment where UHI undertook activities
Nicaragua	Meuwissen et al. 2006 [25]	Voucher scheme	Knowledge of contraceptives, contraceptive use method of preference factors that influence use of contraceptives	• The mean number of problems presented was 1.5 per consultation: 34 % of the vouchers were used for contraceptives, 31 % for complaints related to sexually transmitted infection (STI) or reproductive tract infection (RTI), 28 % for advice/counselling, 28 % for antenatal check-up and 18 % for pregnancy testing. • A new category of health care users emerged: sexually active girls who were neither pregnant nor mothers and who sought contraceptives or STI/RTI treatment. • Contraceptive use doubled among the sexually active non-pregnant voucher redeemers. • Consultation with a female doctor younger than 36 years was associated with a higher chance of having contraceptives prescribed
Uganda & Kenya	Arur et al., 2009 [26]	Voucher scheme	Use, responsiveness, and quality of FP	• In Kenya, uptake of RH-OBA SM vouchers has been high. Between June 2006 and October 2008, 78,651 SM vouchers were sold and 60,581 women used SM vouchers to deliver in a participating facility. • In contrast, use of FP vouchers was considerably lower than expected. In the same period, only 25,620 FP vouchers were sold, and 11,296 (41 %) of these were used. • Examination of provider claims data reveals that FP voucher users overwhelmingly prefer implants to other long-acting and permanent methods. Almost two-thirds (60 %) of FP voucher users selected implants, compared to a third (35 %) who chose female sterilization (bilateral tubal ligation, or BTL). Only 5 % opted for intrauterine contraceptive devices (IUCDs). • Voucher utilization patterns indicate that the poor in Kenya prefer to use private for-profit and non-profit providers. In the area of FP, non-profit providers were the preferred provider (59 %) across all voucher site locations. • Private non-profit providers appear to be a particularly important source of surgical methods of contraception: private non-profit providers submitted 90 % of all claims for BTLs. Non-profit providers were also the preferred provider for SM services and accounted for 45 % of SM claims. • Between February 2009 and June 2009, 4,034 RHVP SM vouchers were sold and close to 2,451 (61 %) used for ANC, institutional deliveries, or PNC services. • Uptake in the first few months of RHVP may have been low as voucher systems take a long time to set up, particularly on the large scale of the RHVP. However, the gap (61 %) between the number of vouchers sold and used is now closing.
Nigeria	Krenn et al. 2014 [22]	Nigerian Urban Reproductive Health Initiative	Awareness and utilisation of FP services	• Between baseline and midterm, the percentage of women who believed in myths or had misconceptions about contraception declined between 9 and 17 % points on outcomes measured • Intention to use contraception in the next 12 months increased between 7.5 and 10.2 % points in four cities • Actual contraception use increased between 2.3 % points (in Abuja) and 15.5 % point (in Kaduna) from baseline to midterm • Reported exposure to several of the Nigerian Urban Reproductive Health Initiative (NURHI) communication interventions was significantly associated with higher levels of contraceptive use. • Propensity score matching found a 9.9 % point increase in contraceptive use in the 4 cities attributable to project exposure
Ghana	Henry et al., 2020 [24]	The Willows home-based counselling and referral programme	Women reported contraceptive use	• 10.5 % point increase in use of modern contraceptives from baseline to close (95 %CI : 6.2, 14.8; *P* < 0.001) and a 7.6 % point increase from baseline to end of project (95 %CI : 3.3, 11.9; *P* < 0.001). • Only 20.2 % of women in the Willows intervention area reported a visit. The intervention, therefore, did not achieve its aim to reach all reproductive-aged women in the community. • The programme had a significant impact on modern contraceptive use at the close of the programme among women who received an information or counselling visit
Bangladesh	Uddin et al., 2012 [23]	Clinics near the place of residence (static clinic and satellite clinics)	Use of family-planning methods among street-dwellers	• The use of healthcare services by the street-dwellers increased at endline compared to baseline in both the model clinic areas, and the difference was highly significant (*p* < 0.001). • Institutional delivery among the female street- dwellers increased at endline compared to baseline in both the clinic areas. • The use of family-planning methods among females also significantly (*p* < 0.001) increased at endline compared to baseline in both the areas.

## Discussion

### Summary of findings

This review aimed to identify existing interventions and service delivery models for providing FP and contraceptive services to slums as well as examine actual and potential impact of past and current interventions and service delivery models in improving access to FP and contraceptive services among slum dwellers in low- and middle-income settings. Overall, only 9 studies and/ or reports were eligible and included in the review, further emphasizing the limited nature of studies on this topic. On the existing service delivery models and interventions for FP services in slums, the review identified six FP service delivery models. These service delivery models were reported from only 9 low- and middle-middle income countries, including Senegal, Uganda, Kenya, Nicaragua, Bangladesh, Pakistan, Ghana, India and Nigeria. Of the six service delivery models, the Urban Reproductive Health Initiative and the voucher scheme service delivery models were the most predominant. As regards the impact of the above-mentioned FP service delivery models in urban slums, the review identified that these interventions hold great promise in significantly improving uptake of FP services. For instance, evidence from this scoping review showed that the identified service delivery models for FP enhanced targeting of poor urban populations, improved efficiency in delivery of FP service, and increased utilization of FP services.

### Findings compared to previous studies

Despite increased research and policy interest in the growing populations in poor urban slums across major cities in low- and middle-income countries, there is limited information on the range of FP service delivery models or interventions. For example, many studies exist on improving access to FP services in both rural and urban populations [30, 31], but most have paid little attention to FP uptake in urban slums. Of the few reviews on this topic, much attention has been focused on evaluation of the voucher scheme in improving uptake and utilization [32, 33].

Evidence from the review has also shown that the use of voucher schemes was highly impactful in terms of improving access to FP services among marginalized groups, a finding consistent with previous studies [32, 34]. This is unsurprising given the fact that the voucher scheme model is highly favoured in improving access to general healthcare services [34]. The use of the voucher scheme as a FP service delivery model suggests a general trend in improved access and uptake of FP services in both poor urban slums and non-slum communities. However, previous studies have highlighted the challenge of targeting beneficiaries in such schemes using validated tools to ensure the most appropriate and deserving beneficiaries have access to FP services [25]. Evidence from other studies have presented important lessons which should guide policy makers and funding agencies in the implementation of policies such as the voucher schemes in urban slums for optimal results.

It is also apparent from the review that, despite the range of FP service delivery models, a number of contextual factors mediate access to such voucher schemes, including cost of FP services, educational level of slum populations as well as cultural norms and values (Stephenson & Hennink, 2004). Thus, efforts should be made to ensure that these services are available and barriers impeding uptake are addressed to reduce the huge unmet needs of populations in slums. The use of vouchers, benefit cards or social franchising schemes are not new within the context of facilitating uptake of FP services. Previous studies have shown that these schemes have been successful in improving uptake of FP services in non-slums areas [32, 33, 35–38]. However, Evidence of a lasting effect is absent, partly because voucher schemes are funded externally and relatively of short duration.

The findings regarding improved access and utilization of FP services in slum communities using the franchised clinics as a service delivery model are consistent with evidence reported from non-slum settings. For example, findings from a Kenyan study showed that the use of franchised clinics resulted in improved access and use of long-acting or permanent methods of contraception [39]. However, the same study did not find increase in access and uptake of other FP services, a finding which questions the widespread applicability of the franchising model for FP services in the general population. It also highlights a need to explore and consider potential facilitating factors promoting the use of FP services through the franchising model. Similar to a study in Pakistan on social franchising where there was remarkable improvement in knowledge on FP services and fulfilment of the unmet needs of people living in urban slums, evidence from Marie Stopes International on a private sector-led social franchising scheme for FP indicated an improvement in access to FP services, choice and quality of FP services [37]. Social franchising in slums may however be limited by the likelihood that few private practitioners are willing to locate in slums. It is also important to note that the availability of such FP service delivery models does not necessarily guarantee uptake, hence factors impeding access should be considered to ensure optimal uptake of available service delivery models. It is therefore important to account for all these contextual factors in order to ensure success in the implementation of the various service delivery models for FP services targeting slums in the urban centres.

Although different activities and strategies were adopted in the urban health initiatives for FP, it was clear from this review that the education and awareness creation initiatives, including the use of posters, billboards, engaging religious and traditional leaders, and the media helped in improving knowledge levels on FP services and overall increase in FP service utilization. Similarly, some previous studies have adopted the media, religious and traditional leaders to improve FP knowledge and utilization, underscoring the potential impact of such approaches in efforts to improve access and utilization of FP services.

### Implications for research and practice

The findings from the present review has important implications for policy makers, governments, NGOs and donors/funders interested in improving access to FP services in poor urban areas and slums. First, the review has advanced our understanding on the different service delivery models and interventions for improving access to FP services in urban slums in low- and middle-income countries. It also provided insights on the potential impact of existing service delivery models for improving access to FP services in slums. For example, the evidence showed that the voucher scheme has potential to improve access and uptake of FP services among populations in slums [26]. These findings could therefore assist policy makers and funding agencies to select the most impactful and potentially impactful service delivery models to improve access and use of FP services in urban slums. While some studies have sought to evaluate the quality of FP services based on specific types of service delivery models, none of the studies in the present review evaluated the quality of FP services provided in urban slums.

Based on this review, we recommend that full systematic review is conducted to evaluate the evidence about the potential impact of each existing service delivery model for FP services in urban slums. Although emerging evidence on the impact of service delivery models were reported in this scoping review, this is less optimal. To establish definitive evidence on the extent of impact of the various service delivery models, future studies should be restricted to experimental and controlled studies. Future studies should also endeavour to add to existing knowledge on the experiences and lessons learnt from implementation of the various FP service delivery models. This information is critical to inform and guide policy makers and funding agencies in the implementation of similar projects which target urban slums. Studies may also take interest in examining the implementation of such service delivery models based on the sex, educational status, employment status, and age distribution of populations in urban slums. We further call for more research/interventions past or current to be published for the purpose of wider learning. Evidence on the quality, health outcomes and efficiency of the variety of service delivery models was scant and thus, this should take priority in future research activities. Importantly, the identified six family planning interventions and service delivery models to improve access to FP in urban slums were reported from different countries with different contexts, cultural values and family planning policies. There is no doubt these factors influenced the extent of implementation of these interventions. It is therefore important to take this into account in any attempt to apply or implement these models in other countries.

### Study limitations and strengths

The review acknowledges certain pertinent limitations. First, although evidence about existing service delivery models for FP in urban slums are presented, including their extent of potential impact, this review did not appraise the methodological rigour of the individual studies given the inclusion of reports (grey literature). Future studies should consider undertaking systematic reviews with priority given to experimental study designs in order to measure the actual impact and effectiveness of the range of FP service delivery models and/ or interventions documented in this study. Assessment of the study findings as reported by the authors was largely relied upon. Policy makers contemplating the use of any of the identified models of care should therefore do so with caution. Further, the findings reported herein were based on the identified peer-reviewed and grey literature using well-defined and broad search terms. Given that the search was restricted to six electronic databases, and English language, it is possible some relevant articles and reports were still missed. Again, there are potentially other contraceptive methods that may have been left out. For instance, some authors refer to bilateral tubal ligation, vasectomy and intrauterine contraceptive device (IUCD), synonymous with IUD, which is included in our search strategy. Articles or reports yet to be reported and not published online could also have been missed, thus minimizing the extent of the evidence. In this review, all intervention designs had some effect on FP uptake, thus demonstrating the scope for improvement in slums populations. But no study examined cost-benefit or sustainability which is an important limitation worth noting.

In spite of the above limitations, some strengths are notable. First, classical of a scoping review approach, this review adopted a broad approach in considering eligible studies for the review. In other words, the eligibility criteria were quite open and flexible and thus allowed for the inclusion of all potentially relevant studies, thus maximizing the final results. Relatedly, the outcomes variables examined for potential effects of the service delivery models were also inclusive and less restrictive.

## Conclusions

Despite increased policy and research interest in improving access to FP services, service delivery models and interventions to improve uptake in urban slums remain limited. The present review revealed 9 eligible studies reporting 6 different service delivery models and/ or interventions for improving uptake of FP services in urban slums. Most of the included studies cited the voucher scheme as an intervention to improve utilization of FP services in urban slums. Thus, the use of voucher system to improve access remains the predominant service delivery model implemented across different countries. This review has identified potential opportunities to inform future research activities to generate more expansive and definitive evidence especially on the potential effects of the different service delivery models. Further efforts are needed to test the efficacy of such interventions using controlled study designs.

## Supplementary Information

**Additional file 1. **MEDLINE Search Strategy.

## Data Availability

All relevant data are included in this paper.

## Abbreviations

ICAS: Central American Health Institute
FP: Family Planning
NGOs: Non-Governmental organisations
NURHI: Nigerian Urban Reproductive Health Initiative
MAG: Married adolescent girls
IUD: Intrauterine Device
RHVP: Reproductive Health Voucher Project
RH-OBA: Reproductive-Health Output-Based Approach
SDGs: Sustainable Development Goals
STIs: Sexually Transmitted Infections
UN: United Nations
