# Supplementary material for: Family planning for urban slums in low- and middle-income countries: a scoping review of interventions/service delivery models and their impact
Source: Int J Equity Health. 2021 Aug 19;20:186. doi: 10.1186/s12939-021-01518-y (PMC8375135; doi:10.1186/s12939-021-01518-y)
Supplement: Supplementary file 1 — Additional file 1. MEDLINE Search Strategy. [file 12939_2021_1518_MOESM1_ESM.docx]

**Supplementary file 1:** MEDLINE Search Strategy

| Search | Query |
| --- | --- |
| 1 | (Family Planning Services) OR (Antenatal Care) OR (Modern Contraception) OR (Abortion) OR (Contraceptives) OR (Condom) OR (Emergency Obstetric Services) OR (Sexuality) OR (Adolescent) OR (Reproductive Health Services) OR (Birth Control) OR (Pills) OR (Intrauterine Device) OR (IUD) OR (Emergency Contraception) OR (Sterilization) OR (Spermicide) OR (Diaphragm) OR (Cervical Cap) OR (Contraceptive Implant) |
| 2 | (Interventions) OR (Programs) OR (Programmes) OR (Service delivery models) OR (Strategies) OR (Policies) OR (Model) OR (Policy) |
| 3 | (Slums) OR (Urban Poor) OR (Poor communities) OR (Downtown) OR (Informal settlements) OR (Unregulated settlements) OR (Favelas) OR (squalid) OR (Overcrowded) OR (ghetto) OR (impoverished areas) OR (Squalid locales) OR (Substandard housing) OR (unplanned settlements) OR (Hovel) OR (sub-standard housing) OR (shanty houses) OR (Overcrowded **homes) OR** (squalid overcrowded house) OR (urban poverty pockets) OR (low-income settlements) OR (squatter settlements) |
| 4 | (Developing country) OR (Low income country) OR (Low-middle income country) OR (Middle income country) OR (Africa) OR (Africa South of the Sahara) OR (Sub-Saharan Africa ) OR (Central Africa) OR (Southern Africa) OR (Easstern Africa ) OR (Western Sahara) OR (East Africa ) OR (Central African Republic ) OR (West Africa ) OR (Cameroon) OR (Chad) OR (Congo) OR (Democratic Republic of Congo) OR (Congo, Demographic Republic) OR (Congo, Republic) OR (Equatorial Guinea) OR (Gabon) OR (Burundi) OR (Djibouti) OR (Eritrea ) OR (Ethiopia ) OR (Kenya) OR (Rwanda) OR (Somalia) OR (Sudan) OR (Tanzania) OR (Uganda) OR (Angola) OR (Botswana) OR (Lesotho) OR (Malawi) OR (Mozambique) OR (Namibia) OR (Swaziland) OR (Zambia) OR (Zimbabwe) OR (Benin) OR (Burkina Faso) OR (Cape Verde) OR (Cote D'ivoire) OR (Gambia) OR (Gambia, The) OR (Ghana) OR (Guinea) OR (Guinea-Bissau) OR (Liberia) OR (Mauritania) OR (Niger) OR (Nigeria) OR (Senegal) OR (Sierra Leone) OR (Togo) OR (South Sudan) OR (Madagascar) OR (Comoros) OR (Mauritius) OR (Sao Tome and Principe) OR (Seychelles) OR (South Africa) |
| 5 | 1 AND 2 AND 3 |
| 6 | 4 AND 5 |
| Limiters | 2000-2020  Full Text  English Language  Academic/Peer reviewed Journals  Humans |
